# Supplementary material for: Use of Capsid Integrity-qPCR for Detecting Viral Capsid Integrity in Wastewater
Source: Viruses. 2023 Dec 26;16(1):40. doi: 10.3390/v16010040 (PMC10819219; doi:10.3390/v16010040)
Supplement: Supplementary file 1 [file viruses-16-00040-s001.zip › viruses-2756990-supplementary.pdf]

Table S1. Primers, probes, and standard sequences used in this study.

Target	Postion	Ref	Primer sequence	Dye/quencher	Target sequence
**Adenovirus**	21418-21555	[1]	Forward: 5'-CATGACTTTTGAGGTGGATC-3' Reverse: 5'-CCGGCCGAGAASGGYGTGCG-CAGGTA-3'	SYBR	CATGACTTTTGAGGTG-GATCCCATGGATGAGCCCACCCTGCTTTATCTTCTTTTCGAAGTCTTCGACGTGGTCAGAGTG-CACCAGCCACAC-CGCGGCGTCATCGAGGCCGTCTACCTGCGCACACCGTTCTCGGCCGG
**Enterovirus**	5'UTR	[2]	Forward: 5'-TGTCACCATAAGCAGYCN-3' Reverse: 5'-CCCTGAATGCGGCTAAT-3' Probe: 5'-ACGGACACCCAAAGTAG-TCGGTTC-3'	HEX-BHQ1	TTAATTGTCACCATAAGCAGCCAATACAA-GAATAAAAAGAAACAC-GGACACCCAAAGTAG-TCGGTTCCGCTGCAGAGTTGCCCGTTACGACACACTGCCCTTTGGATTAAGGGTATGTGCTCCG-CAGTTAGGATTAGCCGCATTCAGGGGCCGGA
**Hepititis-A**	5'UTR	[3]	Forward: 5'-TCACCGCCGTTTGCCTAG-3' Reverse: 5'-GGAGAGCCCTGGAAGAAAG-3' Probe: 5'-TTRATTCCTGCAGGTTCAGG-3'	HEX-BHQ1	TACCTCACCGCCGTTTGCCTAGGC-TATAGGCTAAATTTTCCCTTTCCCTTTTCCCTTTCCTATTCCCTTTGTTTTGCTTGTAAATATTGAT-TTGTAAATATTGATTCCTG-CAGGTTCAGGGTTCTTAAATCTGTTTCTCTATAAGAACACTCATTTCAC-GCTTTCTGTCTTCTTTCTTCCAGGGCTCTCCCCTTG
**Influenza-A**	Segment 7 matrix protein 2	[4]	Forward: 5'-CAAGACCAAT-CYTGTCACCTCTGAC-3' 5'-CAA-GACCAATYCTGTCACCTYTGAC-3' Reverse: 5'-GCATTYTGGACAAA-VCGTCTACG-3' 5'-GCATTTTGGA-TAAAGCGTCTACG-3' Probe 5'-TGCAGTCCT//CGCTCACTGGG-CACG-3'	FAM	CTAAAGACAAGACCAATTCTGTCAC-CTCTGACTAAGGGGATTTTGGGGTTT-GTGTTCAC-GCTCACCGTGCCCAGTGAGCGAGGACTGCAGCGTAGACGCTTTGTCCAAAA
**Influenza-B**	Segment 8 non-structural protein 1	[4]	Forward: 5'-TCCTCAAYTCAC-TCTTCGAGCG-3' Reverse: 5'-CGGTGCTCTTGACCAAATTGG-3' Probe: 5'-CCAATTCGA//GCAGCTGAAACTGCGGTG-3'	YAK	GGATCCTCAACTCACTCTTCGAGCGTTTT-GATGAAGGACATTCAAAGCCAATTCGAG-CAGCTGAAACTGCGGTGGGAGTCTTATCCCAATTTGGTCAAGAGCACCGATT
**Noro GI**	ORF1-RdRp	[3]	Forward: 5'-CGCTG-GATGCGNTTCCAT-3' Reverse: 5'-CCTTAGACGCCATCATCATTTAC-3' Probe: 5'-TGGACAGGAGATCGC-3'	FAM-MGB	CGCTGGATGCGATTCCATGACTTAAGTTTGTG-GACAGGAGATCGCGATCTCTTGCCCGAT-TATGTAAATGATGATGGCGTCTAAGG
**Noro GII**	ORF1-RdRp	[3]	Forward: 5'-ATGTTCAGRT-GGATGAGRTTCTCWGA-3' Reverse 5'-TCGACGCCATCTTCATTCACA-3' Probe: 5'-AGCACGTGG-GAGGGCGATCG-3'	DO-DDQII	ATGTTCAGATGGATGAGATTCTCAGATCTGAG-CACGTGGGAGGGCGATCG-CAATCTGGCTCCCAGTTTTGTGAATGAAGATGGCGTCGA
**SARS-CoV-2**	N1	[5]	Forward: 5'-GACCCCAAAATCAGCGAAAT-3' Re-verse: 5'- TCTGGTTACTGCCAGTT-GAATCTG-3' Probe: 5'-ACCCCG-CATTACGTTTGGTGGACC-3'	FAM-MGB	GACCCCAAAATCAGCGAAATGCACCCCG-CATTACGTTTGGTGGACCCTCAGAT-TCAACTGG-CAGTAACCAGAATGGTGGGGCGCGATCAAAACAACGTCGGCCCCA

References

1. Van Maarseveen, N.M.; Wessels, E.; De Brouwer, C.S.; Vossen, A.C.T.M.; Claas, E.C.J. Diagnosis of Viral Gastroenteritis by Simultaneous Detection of Adenovirus Group F, Astrovirus, Rotavirus Group A, Norovirus Genogroups I and II, and Sapovirus in Two Internally Controlled Multiplex Real-Time PCR Assays. *Journal of Clinical Virology* **2010**, *49*, 205–210, doi:10.1016/j.jcv.2010.07.019.

2. Gregory, J.B.; Litaker, R.W.; Noble, R.T. Rapid One-Step Quantitative Reverse Transcriptase PCR Assay with Competitive Internal Positive Control for Detection of Enteroviruses in Environmental Samples. *Appl Environ Microbiol* **2006**, *72*, 3960–3967, doi:10.1128/AEM.02291-05.

3. ISO 15216-2:2019 ISO 15216-2:2019 - Microbiology of the Food Chain — Horizontal Method for Determination of Hepatitis A Virus and Norovirus Using Real-Time RT-PCR — Part 2: Method for Detection Available online: https://www.iso.org/standard/74263.html (accessed on 6 November 2023).

4. Shu, B.; Kirby, M.K.; Davis, W.G.; Warnes, C.; Liddell, J.; Liu, J.; Wu, K.H.; Hassell, N.; Benitez, A.J.; Wilson, M.M.; et al. Multiplex Real-Time Reverse Transcription PCR for Influenza A Virus, Influenza B Virus, and Severe Acute Respiratory Syndrome Coronavirus 2 - Volume 27, Number 7—July 2021 - Emerging Infectious Diseases Journal - CDC. *Emerg Infect Dis* **2021**, *27*, 1821–1830, doi:10.3201/EID2707.210462.

5. Lu, X.; Wang, L.; Sakthivel, S.K.; Whitaker, B.; Murray, J.; Kamili, S.; Lynch, B.; Malapati, L.; Burke, S.A.; Harcourt, J.; et al. US CDC Real-Time Reverse Transcription PCR Panel for Detection of Severe Acute Respiratory Syndrome Coronavirus 2. *Emerg Infect Dis* **2020**, *26*, 1654, doi:10.3201/EID2608.201246.
